# From Respiratory Pathogen to Systemic Threat: Rethinking *Mycoplasma pneumoniae* Infections

**DOI:** 10.3390/microorganisms14020419

**Published:** 2026-02-10

**Authors:** Marco Bongiovanni

**Affiliations:** 1 Division of Infectious Diseases, Ente Ospedaliero Cantonale, 6900 Lugano, Switzerland; marco.bongiovanni@eoc.ch; 2 Department of Biomedical Sciences, University of Southern Switzerland, 6900 Lugano, Switzerland

**Keywords:** *Mycoplasma pneumonia*, extrapulmonary manifestations, systemic disease

## Abstract

*Mycoplasma pneumoniae* is traditionally recognized as a leading cause of community-acquired pneumonia, yet growing evidence demonstrates that its clinical impact extends far beyond the respiratory tract. Increasing reports of neurologic, cardiac, hematologic, dermatologic, renal, gastrointestinal, and thrombotic complications indicate that *M. pneumoniae* should be viewed as a systemic pathogen capable of inducing multisystem disease. Extrapulmonary manifestations may arise through three major mechanisms: direct bacterial invasion of tissues, immune-mediated injury driven by molecular mimicry or immune complexes, and vascular or thrombotic events related to endothelial dysfunction. These processes frequently occur independently of, or temporally dissociated from, respiratory symptoms, complicating early diagnosis. The diagnostic approach remains challenging because respiratory PCR may reflect colonization, serology is delayed, and pathogen detection in sterile sites is uncommon. Consequently, diagnosis often depends on the integration of clinical features, laboratory markers, and organ-specific imaging. Management requires a combined strategy: antimicrobial therapy to reduce bacterial load, organ-targeted supportive measures, and immunomodulatory interventions such as corticosteroids, IVIG, or plasma exchange for severe immune-mediated complications. The emergence of macrolide-resistant strains further underscores the need for tailored antimicrobial strategies and close clinical monitoring. Although many extrapulmonary complications are reversible, severe forms—including encephalitis, ADEM, myocarditis, Stevens–Johnson syndrome, and major thromboses—can lead to lasting morbidity or death. Significant knowledge gaps persist, including determinants of host susceptibility, mechanisms linking CARDS toxin to systemic inflammation, the impact of macrolide resistance on disease severity, and the absence of standardized diagnostic criteria. Advances in molecular immunology, multicenter registries, and development of targeted therapies or vaccines represent crucial next steps. Overall, the breadth and clinical relevance of extrapulmonary involvement support a paradigm shift: *Mycoplasma pneumoniae* infection should be regarded and managed as a systemic disease rather than a purely respiratory pathogen.

## 1. Introduction

*Mycoplasma pneumoniae* is an atypical bacterial pathogen characterized by the absence of a cell wall and a reduced genome, making it one of the most biologically unique and complex respiratory pathogens. First identified in the 1940s as a cause of epidemic pneumonia in children, *M. pneumoniae* was initially studied primarily in the context of respiratory disease, manifesting as a variable presentation of community-acquired pneumonia—commonly termed “atypical” due to mild symptoms and discrepancies between clinical features and radiographic findings [1,2]. While traditionally associated with pulmonary involvement, over the past decades there has been increasing recognition of *M. pneumoniae* as a cause of systemic disease, affecting virtually every major organ system. These extrapulmonary manifestations are now recognized as clinically significant and can pose diagnostic and therapeutic challenges [3,4].

*M. pneumoniae* has a wide global distribution, with periodic outbreaks occurring approximately every 3–7 years, although sporadic cases are observed year-round [5,6,7,8,9,10,11]. The true burden of disease remains difficult to estimate because asymptomatic carriage and mild or subclinical infection are common, diagnostic testing is heterogeneous across studies, and extrapulmonary syndromes often present after respiratory symptoms have resolved. Available data suggest that extrapulmonary manifestations occur in a minority of infected individuals. In pediatric hospital-based cohorts, extrapulmonary complications have been reported in up to 10–20% of symptomatic respiratory infections, whereas population-based surveillance studies often report lower proportions, sometimes below 5%. Most frequently documented complications include cold agglutinin autoimmune hemolytic anemia, *M. pneumoniae*-induced rash and mucositis (MIRM), neurological syndromes such as encephalitis, acute disseminated encephalomyelitis (ADEM), and mild encephalitis/encephalopathy with reversible splenial lesion (MERS), as well as immune-mediated myocarditis or pericarditis. Less common manifestations, including thrombotic events, renal syndromes, and rheumatologic disorders, are primarily reported in case series or isolated case reports. Although infrequent, these complications can carry disproportionate clinical impact due to their potential severity. Age-specific patterns are observed: children and adolescents are more prone to neurological and mucocutaneous complications, whereas adults more frequently develop cardiac, thrombotic, or hematologic manifestations. Environmental and epidemiological factors—including seasonality (often peaking in autumn and winter), school-based transmission, and international travel—also influence both incidence and outbreak dynamics.

The pathogenic success of *M. pneumoniae* is partly due to its virulence mechanisms and host interactions [12,13]. Surface adhesins enable persistent attachment to respiratory epithelial cells, while the Community-Acquired Respiratory Distress Syndrome (CARDS) toxin has been shown to induce robust inflammatory responses. Recent studies indicate that CARDS toxin can trigger systemic pro-inflammatory cytokine release and immune activation, potentially contributing to extrapulmonary pathology [14,15,16]. In parallel, autoantibody formation and immune complex deposition suggest a central role of immune-mediated mechanisms, including molecular mimicry and transient autoimmune phenomena [17,18,19].

Three possible mechanisms at the basis of extrapulmonary manifestations have been postulated: (1) a direct type in which the bacterium is present at the site of inflammation, and local inflammatory cytokines induced by the bacterium play a key role; (2) an indirect type in which the bacterium is not present at the site of inflammation and immune modulations, such as autoimmunity or the formation of immune complexes, play a role; (3) a vascular occlusion type in which obstruction of blood flow either directly or indirectly caused by the bacterium plays a crucial role [16].

From a clinical perspective, extrapulmonary manifestations of *M. pneumoniae* present substantial diagnostic challenges. In many cases, respiratory symptoms are mild, transient, or absent, and systemic complications may occur days to weeks after acute infection. This temporal dissociation complicates early recognition and can delay targeted therapy. Laboratory diagnosis is also limited: respiratory PCR is sensitive but may reflect colonization or prior infection; PCR in sterile body fluids such as cerebrospinal fluid (CSF) is often negative even in clinically confirmed encephalitis; and serology requires time to detect IgM/IgG responses. Therefore, clinicians must integrate clinical suspicion, laboratory findings, imaging, and immunologic markers to establish a diagnosis.

In recent years, the emergence of macrolide-resistant *M. pneumoniae* strains in certain regions has further complicated disease management, highlighting the need for alternative antibiotics and, in severe extrapulmonary cases, immunomodulatory strategies. Moreover, the literature emphasizes the importance of a multidisciplinary approach—pediatrics, infectious diseases, neurology, cardiology, dermatology, and hematology—to optimize diagnosis, treatment, and prognosis.

This narrative review aims to provide an up-to-date and comprehensive synthesis of *M. pneumoniae* extrapulmonary complications, including epidemiology, pathogenesis, organ-specific presentations, diagnostic approaches, therapeutic strategies, and knowledge gaps. Our goal is to offer a practical clinical guide while highlighting areas for future research on this versatile and potentially severe pathogen.

## 2. Literature Search

For this narrative review, we performed a comprehensive literature search to summarize current knowledge on extrapulmonary manifestations of *Mycoplasma pneumoniae*. Relevant studies published from 1980 to 2025 were identified through PubMed/MEDLINE, Scopus, and Web of Science using combinations of keywords including “Mycoplasma pneumoniae,” “extrapulmonary,” “neurological,” “cardiac,” “hematologic,” “dermatologic,” and “vascular.” Articles were selected based on clinical relevance, quality of data, and the presence of microbiologic confirmation or a clear temporal association with respiratory infection. Additional references were identified through cross-referencing of key articles.

## 3. Organ-System Presentations and Clinical Complications

Extrapulmonary manifestations of *M. pneumoniae* can potentially affect nearly every organ system (Table 1). These complications often occur independently of, or after, the respiratory illness and may present as isolated syndromes, complicating early recognition. Clinical heterogeneity arises from a combination of direct bacterial invasion, immune-mediated injury, and vascular or thrombotic phenomena. Understanding the clinical spectrum, pathophysiology, and management strategies for each organ system is essential for timely diagnosis and intervention.

### 3.1. Evidence Prioritisation: Syndromes Most Strongly Associated with M. pneumoniae

Although *M. pneumoniae* has been reported in association with a wide range of extrapulmonary syndromes, the strength of evidence is not uniform across manifestations. The most consistently supported complications—based on repeated clinical observations, biological plausibility, and supportive laboratory findings—include: (i) cold agglutinin autoimmune haemolytic anaemia; (ii) mucositis and erythema multiforme spectrum disease (including Mycoplasma-induced rash and mucositis, MIRM); (iii) selected central nervous system syndromes such as encephalitis/encephalopathy including MERS and post-infectious demyelinating disease (e.g., ADEM); and (iv) myocarditis/pericarditis in temporal association with respiratory infection. In contrast, other reported manifestations (e.g., some rheumatologic syndromes, vasculitic presentations, or rare endocrine/ocular events) are supported mainly by case reports and small series, and causality is more difficult to establish. For this reason, throughout this review we aim to distinguish well-supported complications from those where the association remains plausible but less definitive.

### 3.2. Neurological Complications

Neurologic involvement represents one of the most clinically significant extrapulmonary manifestations of M. pneumoniae due to its potential for morbidity and long-term sequelae. Reported syndromes include meningoencephalitis, acute disseminated encephalomyelitis (ADEM), transverse myelitis, brainstem encephalitis, cerebellitis with ataxia, cranial neuropathies (including optic neuritis), movement disorders such as chorea, and peripheral neuropathies including Guillain–Barré syndrome (GBS) and its variants [20,21,22,23,24,25,26,27,28]. Neurological symptoms can develop acutely or subacutely, typically within days to weeks after respiratory infection, though they may occasionally precede pulmonary involvement. Importantly, while an association between *M. pneumoniae* and GBS has been reported, the evidence is largely observational and causality is not always demonstrable; therefore, alternative infectious triggers should be actively considered and excluded when evaluating post-infectious neuropathy.

The pathophysiology of neurological complications is largely immune-mediated, although rare cases of direct bacterial invasion of the central nervous system have been documented. Molecular mimicry, immune complex deposition, and cytokine-driven neuro-inflammation are implicated in syndromes such as ADEM and GBS. Imaging studies, particularly MRI, are crucial for diagnosis; findings may include multifocal demyelinating lesions, diffuse T2 hyperintensities, or transient splenial lesions characteristic of MERS (mild encephalitis/encephalopathy with reversible splenial lesion) [29,30]. CSF analysis typically shows lymphocytic pleocytosis and elevated protein, but PCR detection of *M. pneumoniae* in CSF is uncommon [31]. Early recognition and intervention, including macrolide therapy, corticosteroids, IVIG, or plasma exchange, are associated with improved outcomes, though severe disease can result in permanent deficits or death. Long-term follow-up is often needed to assess recovery, particularly for cognitive or motor sequelae.

### 3.3. Cardiovascular Complications

Cardiac involvement in *M. pneumoniae* infection encompasses myocarditis, pericarditis, myopericarditis, arrhythmias, and, rarely, intracardiac thrombi or endocarditis [32,33,34,35,36,37,38]. Clinical presentations range from mild chest discomfort or palpitations to fulminant myocarditis with heart failure, cardiogenic shock, or sudden arrhythmic death, particularly in adolescents and young adults. Pathogenesis may involve direct myocardial invasion in some cases, immune-mediated injury with autoantibody production, and cytokine-induced myocardial inflammation. Cardiac biomarkers (troponin, BNP) and electrocardiography aid initial assessment, while echocardiography and cardiac MRI allow evaluation of ventricular function, inflammation, and fibrosis. Myocardial biopsy is rarely performed but can provide definitive evidence of inflammatory infiltration or bacterial presence. Management combines supportive care for heart failure, arrhythmia monitoring, antimicrobial therapy, and immunomodulation when immune-mediated myocarditis is suspected. Outcomes depend on early recognition; severe or fulminant myocarditis carries a high risk of morbidity and mortality, while milder forms are often reversible with appropriate therapy.

### 3.4. Haematologic Complications

*M. pneumoniae* can induce a spectrum of hematologic disorders, the most common of which is cold agglutinin autoimmune haemolytic anemia (cAIHA). This condition is mediated by IgM antibodies that agglutinate red blood cells at low temperatures, leading to complement-mediated haemolysis [39,40]. Clinically, patients may present with pallor, jaundice, haemoglobinuria, and fatigue. Laboratory findings include haemolysis markers (elevated LDH, indirect hyperbilirubinemia), positive direct antiglobulin test (DAT), and elevated cold agglutinin titers. Management involves treating the underlying infection, maintaining warmth to reduce haemolysis, and providing transfusions in severe cases. Corticosteroids or other immunosuppressive therapies may be necessary for refractory hemolysis [41,42]. Less common hematologic manifestations include thrombocytopenia, transient antiphospholipid antibody production, and disseminated intravascular coagulation [43,44,45,46]. While these are generally reversible, severe thrombocytopenia or coagulopathy can be life-threatening and require prompt intervention.

### 3.5. Dermatological and Mucocutaneous Disease

Cutaneous manifestations of *M. pneumoniae* infection are varied, ranging from mild maculopapular rashes and urticaria to severe mucocutaneous disorders such as erythema multiforme, Stevens–Johnson syndrome (SJS), and toxic epidermal necrolysis (TEN) [47,48,49,50,51,52]. *M. pneumoniae* induced mucositis, often occurring in children and young adults, is characterized by prominent mucosal involvement with minimal skin lesions, leading to significant oral, ocular, or genital morbidity. Pathogenesis is immune-mediated, with molecular mimicry and cytokine-induced epithelial injury contributing to tissue damage. Management focuses on supportive care, intensive ophthalmologic involvement, and selective systemic immunomodulation using corticosteroids, IVIG, or cyclosporine in severe cases. Early recognition and specialized care are critical to minimize complications such as permanent ocular damage or mucosal scarring.

### 3.6. Musculoskeletal and Rheumatological Complications

Musculoskeletal manifestations of *M. pneumoniae* include myalgias, arthralgias, and, rarely, frank arthritis, which may be reactive or, infrequently, septic [53,54,55,56,57,58]. Rhabdomyolysis has also been reported in isolated cases, sometimes accompanied by acute kidney injury due to myoglobinuria [59,60]. Immune-mediated mechanisms are likely responsible for most musculoskeletal presentations, although direct invasion has been hypothesized in some cases. Symptoms are typically self-limiting but may require analgesia and supportive care. Clinicians should maintain vigilance for persistent joint inflammation, particularly in post-infectious reactive arthritis.

### 3.7. Renal Complications

Renal involvement is uncommon but clinically important. *M. pneumoniae* has been associated with acute glomerulonephritis and other immune-mediated renal syndromes, likely resulting from immune complex deposition or cross-reactive antibodies [61,62,63,64]. Presentations may range from asymptomatic hematuria and proteinuria to nephritic or nephrotic syndromes with renal dysfunction. Laboratory assessment includes serum creatinine, urinalysis, and complement evaluation. Severe renal involvement may necessitate renal biopsy for definitive diagnosis and, rarely, dialysis for acute kidney injury. Management is supportive and includes infection control and immunomodulation in select cases.

### 3.8. Gastrointestinal and Hepatic Complications

Gastrointestinal manifestations are generally nonspecific, including nausea, vomiting, diarrhea, and abdominal discomfort. Rarely, *M. pneumoniae* infection has been associated with acute pancreatitis or clinically significant hepatitis, which may present with right upper quadrant pain, jaundice, and elevated liver enzymes [65,66,67,68]. Hepatic involvement is usually transient and resolves following control of the infection. Recognition is important to avoid misdiagnosis and unnecessary invasive interventions.

### 3.9. Vascular and Thrombotic Complications

*M. pneumoniae* infection can precipitate vascular complications through transient endothelial activation, hypercoagulability, and antiphospholipid antibody production [69,70,71]. Documented events include arterial and venous thromboses, stroke, limb ischemia, splenic or renal infarction, and intra-cardiac thrombi. Thrombotic events are typically acute and may occur in young, otherwise healthy individuals. Laboratory assessment often reveals transient antiphospholipid antibodies, which generally normalize after infection resolution [44]. Management requires anticoagulation for confirmed thrombosis, with follow-up testing to distinguish transient infection-related prothrombotic states from persistent thrombophilia.

## 4. Diagnostic Approach: Practical Considerations and Pitfalls

Extrapulmonary manifestations of *M. pneumoniae* remain diagnostically challenging due to their heterogeneous presentations and frequent dissociation from respiratory symptoms. A high index of suspicion is essential, particularly in children and young adults presenting with neurological, cardiac, hematologic, dermatologic, or thrombotic complications in the context of recent or concurrent respiratory illness. Recognizing subtle respiratory symptoms, such as mild cough or rhinorrhoea, can be crucial in linking systemic findings to a preceding *M. pneumoniae* infection.

Microbiologic confirmation typically relies on PCR testing of respiratory specimens, which remains the most sensitive tool to detect acute infection. Nevertheless, PCR positivity must be interpreted with caution, as it may reflect prior colonization or a recently resolved infection rather than active disease. Therefore, attribution of extrapulmonary pathology to *M. pneumoniae* should rely on a synthesis of temporal association, compatible clinical phenotype, supportive serology (e.g., seroconversion or rising titres), and exclusion of alternative causes. Serology, particularly the detection of rising IgM and IgG titers, can support the diagnosis when PCR is negative or unavailable, although antibody responses often lag behind symptom onset. In cases of neurological involvement, CSF testing is sometimes employed, yet PCR detection rates in CSF remain low, underscoring the importance of integrating clinical, serologic, and imaging evidence when evaluating central nervous system complications.

Organ-specific investigations are guided by clinical presentation. For neurological complications, magnetic resonance imaging of the brain may reveal diffuse or focal lesions, including transient splenial hyperintensities characteristic of mild encephalitis/encephalopathy with reversible splenial lesion (MERS). Cardiac involvement is evaluated through electrocardiography, cardiac biomarkers such as troponin and BNP, and imaging modalities including echocardiography and cardiac MRI. Hematologic assessment includes complete blood counts, peripheral smears, and testing for cold agglutinins or other autoimmune antibodies. Dermatologic manifestations may require skin biopsy and thorough mucosal evaluation, often with ophthalmology input, particularly in suspected Stevens–Johnson syndrome or toxic epidermal necrolysis. For renal or thrombotic complications, laboratory evaluation of kidney function, complement levels, coagulation profiles, and autoantibodies helps clarify the mechanism and guides management.

Interpreting these investigations requires careful clinical judgment. PCR detection in the respiratory tract may indicate asymptomatic colonization rather than causation, and serology can cross-react with other pathogens or be delayed. In practice, most diagnostic approaches rely on a synthesis of clinical features, laboratory evidence, and imaging, rather than a single definitive test. Multidisciplinary assessment is often necessary, especially when multiple organ systems are involved.

## 5. Management Principles and Therapeutic Strategies

Management of extrapulmonary manifestations of *M. pneumoniae* is inherently complex due to the heterogeneous nature of organ involvement, the variable timing of symptom onset relative to respiratory infection, and the interplay of direct bacterial invasion and immune-mediated injury. A comprehensive approach combines targeted antimicrobial therapy, organ-specific supportive measures, immunomodulatory interventions, and close multidisciplinary collaboration. The therapeutic strategy must be individualized, taking into account patient age, comorbidities, severity of systemic involvement, and local antimicrobial resistance patterns.

### 5.1. Antimicrobial Therapy

Antimicrobial therapy remains the cornerstone of *M. pneumoniae* management, aiming to eradicate the pathogen, reduce bacterial load, and potentially mitigate immune-mediated complications by limiting ongoing antigenic stimulation. In paediatric populations, macrolides such as azithromycin or erythromycin are generally first-line due to their efficacy, favourable safety profile, and ability to penetrate respiratory and systemic tissues. In older children and adults, tetracyclines or respiratory fluoroquinolones are considered, provided age-related contraindications and safety concerns are addressed [72] (Table 2).

The emergence of macrolide-resistant *M. pneumoniae* strains, increasingly reported worldwide, complicates therapy [73,74,75]. Resistance may prolong infection, exacerbate systemic immune activation, and delay clinical recovery. Clinicians should monitor for persistent fever, worsening organ-specific symptoms, or lack of response to macrolides, and consider alternative agents or combination therapy guided by susceptibility data when available. Evidence regarding the impact of early antibiotic initiation on the prevention of extrapulmonary complications is limited, but biological plausibility suggests that prompt pathogen eradication could reduce antigenic stimulus and subsequent immune-mediated injury.

Dosing and duration of antimicrobial therapy are influenced by severity of infection, site of involvement, and patient factors. For neurologic or cardiac involvement, some experts recommend extended courses to ensure systemic bacterial clearance, although standardized protocols are lacking. Clinicians must balance the potential benefits of prolonged therapy with the risks of adverse effects, including hepatotoxicity, gastrointestinal intolerance, and selection of resistant organisms.

### 5.2. Immunomodulatory Therapy

Immune-mediated injury is central to many extrapulmonary *M. pneumoniae* manifestations, including acute disseminated encephalomyelitis, Guillain–Barré syndrome, severe muco-cutaneous disease, and immune myocarditis. In such cases, immunomodulatory therapy is a key adjunct to antimicrobial treatment. The choice of immunotherapy depends on the organ system involved, disease severity, and the patient’s immune status.

Corticosteroids are commonly used to reduce inflammatory tissue damage [76,77]. In neurologic complications such as ADEM or fulminant encephalitis, high-dose intravenous corticosteroids can mitigate demyelination and cerebral edema. In severe mucocutaneous disease, corticosteroids may help control widespread inflammation, although their use remains controversial and should be individualized.

Intravenous immunoglobulin (IVIG) is frequently employed in severe neurologic disease, including Guillain–Barré syndrome and Bickerstaff brainstem encephalitis, as well as in fulminant immune-mediated myocarditis. IVIG can modulate pathogenic autoantibodies, suppress pro-inflammatory cytokines, and improve clinical outcomes. Dosing protocols vary but generally involve 2 g/kg administered over two to five days [78,79].

Plasma exchange (plasmapheresis) is indicated in selected severe cases, particularly when autoantibody-mediated processes are suspected, such as in fulminant haemolytic anemia or refractory neurologic syndromes. Plasmapheresis facilitates rapid removal of circulating immune complexes, antibodies, and inflammatory mediators [80].

Emerging therapies targeting specific immune pathways are under investigation, including monoclonal antibodies against key cytokines or immune checkpoints involved in *M. pneumoniae* induced systemic inflammation. While these approaches remain experimental, they hold promise for selective modulation of immune-mediated tissue injury without broadly suppressing host defences.

### 5.3. Organ-Specific Supportive Care

Supportive care is essential and must be tailored to the organ system involved:

Patients with encephalitis, ADEM, or peripheral neuropathy require neurocritical monitoring, seizure control, management of intracranial pressure, and long-term rehabilitation including physical, occupational, and cognitive therapy. Early mobilization and comprehensive rehabilitation can improve functional outcomes.

Management of myocarditis or pericarditis includes standard heart failure therapies (diuretics, ACE inhibitors, beta-blockers as tolerated), arrhythmia monitoring and treatment, and hemodynamic support in cases of cardiogenic shock. Immune-mediated myocarditis may necessitate adjunctive corticosteroids or IVIG. In cases of intra-cardiac thrombi or high-risk arrhythmias, anticoagulation and electrophysiologic interventions may be indicated.

Severe haemolysis due to cold agglutinins requires transfusion support, warming measures to prevent cold-induced haemolysis, and monitoring for secondary complications such as renal dysfunction or cardiovascular strain. Refractory cases may require corticosteroids or other immunosuppressive therapies.

Stevens–Johnson syndrome or toxic epidermal necrolysis necessitates intensive supportive care, often in burn or ICU units, with meticulous wound care, fluid and electrolyte management, and infection prevention. Ophthalmologic consultation is crucial to prevent permanent ocular damage. Systemic immunomodulation is considered on a case-by-case basis.

Acute kidney injury requires fluid management, monitoring of electrolytes, and renal replacement therapy if indicated. Hepatic involvement is generally managed supportively, with careful monitoring of liver function tests and avoidance of hepatotoxic medications.

Documented thrombotic events require anticoagulation with agents selected based on age, renal function, and bleeding risk. In transient infection-associated antiphospholipid states, anticoagulation is continued until laboratory normalization, with follow-up to rule out persistent thrombophilia.

## 6. Adjunctive and Emerging Therapies

Beyond conventional antimicrobial and immunomodulatory approaches, supportive strategies such as physiotherapy, occupational therapy, and nutritional optimization are integral to recovery, particularly in severe neurological or mucocutaneous disease. Novel approaches under investigation include targeted immunotherapies, biologic agents to block specific inflammatory pathways, and CARDS-toxin inhibitors. These therapies aim to minimize immune-mediated damage while maintaining host defences.

Effective management of extrapulmonary *M. pneumoniae* disease is primarily guided by syndrome severity. Most patients require standard antimicrobial therapy and supportive care; however, severe, complicated, or multisystem presentations (e.g., encephalitis, fulminant myocarditis, SJS/TEN, major thrombosis, or refractory haemolysis) benefit from early involvement of relevant specialties. Infectious disease specialists guide antimicrobial therapy, while neurologists, cardiologists, haematologists, dermatologists, nephrologists, intensivists, and rehabilitation teams contribute organ-specific expertise when indicated.

## 7. Prognosis, Knowledge Gaps, and Future Directions

The prognosis of extrapulmonary manifestations of *M. pneumoniae* varies widely depending on the organ system involved, the severity of the complication, and the timeliness of diagnosis and intervention. Many extrapulmonary complications are reversible, particularly in paediatric populations. For example, children with mild neurological syndromes, including transient splenial lesions or post-infectious encephalopathy, often experience full recovery within weeks to months. Similarly, cold agglutinin haemolysis is typically self-limiting, resolving after the acute infection is controlled. Musculoskeletal symptoms and mild gastrointestinal or hepatic involvement generally follow a benign course when appropriately supported.

However, severe complications can result in significant morbidity or mortality. Fulminant encephalitis, acute disseminated encephalomyelitis with prolonged coma, fulminant myocarditis, Stevens–Johnson syndrome or toxic epidermal necrolysis with extensive mucosal involvement, and major thrombotic events can produce long-term deficits, organ dysfunction, or even death. Case reports and series highlight instances of permanent neurological impairment, chronic cardiac sequelae, and visual loss due to severe mucosal damage. Long-term follow-up studies are scarce, limiting our understanding of chronic consequences and recurrence risk.

Several factors appear to influence prognosis. Early recognition and intervention are consistently associated with improved outcomes. The presence of pre-existing comorbidities, delayed initiation of antimicrobial therapy, and high bacterial load may predispose to more severe disease. In immune-mediated complications, rapid initiation of immunomodulatory therapy—such as corticosteroids, intravenous immunoglobulin, or plasma exchange—can alter the clinical course, although the optimal timing, dosing, and duration of these therapies remain incompletely defined.

Despite increasing recognition of *M. pneumoniae* systemic impact, important knowledge gaps persist. The reasons why only a minority of *M. pneumoniae* infections lead to extrapulmonary complications are not fully understood, suggesting a role for host genetic susceptibility, variations in immune response, and pathogen-specific virulence factors [81,82,83,84,85]. The relative contributions of direct bacterial invasion versus immune-mediated injury in specific organ systems remain debated, and the mechanistic pathways linking CARDS toxin activity, autoantibody production, and endothelial dysfunction require further elucidation. Similarly, the influence of macrolide resistance on the development or severity of extrapulmonary manifestations remains an open question, particularly in regions with high resistance prevalence.

Optimal management strategies for immune-mediated complications are also incompletely defined. While corticosteroids, intravenous immunoglobulin, and plasma exchange are widely used, evidence guiding precise regimens, timing, and duration is largely derived from case series rather than randomized controlled trials. Standardized diagnostic criteria for extrapulmonary *M. pneumoniae* disease are lacking, complicating the comparison of studies and the aggregation of outcome data. High-quality prospective studies, multicenter registries, and harmonized case definitions would help clarify incidence, pathogenesis, and optimal therapeutic approaches.

## 8. Conclusions and Future Directions

Despite significant advances in understanding *M. pneumoniae* and its extrapulmonary manifestations, many questions remain unanswered, offering fertile ground for future research. One of the primary challenges is elucidating why only a subset of individuals develop systemic complications. Identifying host genetic and immunologic factors that confer susceptibility could transform our ability to predict and prevent severe disease. Studies exploring variations in immune response, including HLA haplotypes, cytokine profiles, and polymorphisms in innate immunity genes, could clarify why some patients experience robust inflammatory reactions leading to organ-specific pathology while others remain asymptomatic.

Another critical avenue for research involves dissecting the relative contributions of direct bacterial invasion versus immune-mediated injury across different organ systems. While evidence supports both mechanisms, the precise interplay remains unclear. For example, the detection of *M. pneumoniae* DNA in cerebrospinal or synovial fluid is rare, yet immune-mediated syndromes such as ADEM, Guillain–Barré syndrome, and Stevens–Johnson syndrome are common. Advanced molecular techniques, including single-cell transcriptomics and spatial proteomics, may enable the identification of pathogen-host interactions at the tissue level, revealing specific immune pathways responsible for tissue damage. Understanding these pathways could guide the development of targeted immunotherapies that modulate pathological immune responses without broadly suppressing host immunity.

The role of the CARDS toxin in extrapulmonary disease is another promising research frontier. Although this toxin is known to induce pro-inflammatory cytokine release and contribute to pulmonary pathology, its systemic effects are not fully characterized. Investigating how CARDS toxin interacts with endothelial cells, immune cells, and specific organ microenvironments may uncover mechanisms underlying neurological, cardiac, and vascular complications. Furthermore, studies exploring neutralizing antibodies or small-molecule inhibitors against CARDS toxin could provide a foundation for therapeutic interventions that specifically mitigate toxin-mediated damage.

Macrolide resistance represents a growing clinical concern. While the impact of resistant strains on pulmonary outcomes has been relatively well-studied, their influence on extrapulmonary complications is poorly understood. Future studies should examine whether resistant strains provoke prolonged antigenic stimulation or altered immune responses, potentially increasing the risk or severity of systemic manifestations. Epidemiologic surveillance combining microbiologic data with detailed clinical phenotyping could clarify these relationships and influence regional treatment guidelines.

From a clinical perspective, the development of standardized diagnostic criteria and consensus definitions for extrapulmonary *M. pneumoniae* disease is urgently needed. The current reliance on case reports and heterogeneous series limits comparability and the ability to synthesize outcomes. Prospective multicenter registries incorporating comprehensive clinical, laboratory, imaging, and immunologic data could provide a robust framework to determine incidence, risk factors, and prognostic indicators. Integration of advanced imaging techniques, biomarker discovery, and machine-learning algorithms may further enhance early detection and risk stratification, allowing clinicians to identify high-risk patients before severe complications occur.

Therapeutically, the optimal use of immunomodulatory agents remains unclear. Randomized controlled trials comparing corticosteroids, intravenous immunoglobulin, plasma exchange, or combination therapies are lacking. Additionally, research into novel immunotherapies, such as monoclonal antibodies targeting specific cytokines or immune checkpoints involved in *M. pneumoniae*-related inflammation, could provide more precise treatment options with fewer systemic side effects. Investigating the timing, dosage, and duration of these interventions is essential to maximize efficacy while minimizing adverse outcomes.

Vaccine development represents an important long-term preventive strategy. Despite decades of research, no licensed *M. pneumoniae* vaccine is available. Modern approaches—including subunit vaccines targeting adhesins or CARDS toxin, mRNA-based platforms, or live-attenuated strains—warrant further investigation, particularly to reduce disease burden during cyclical outbreaks and potentially prevent severe complications in high-risk groups [86,87,88].

Finally, collaborative, multidisciplinary research is critical. Integration of infectious disease specialists, immunologists, neurologists, cardiologists, haematologists, dermatologists, and epidemiologists will enable comprehensive study designs that address both mechanistic and clinical questions. Such collaborations, coupled with advanced omics technologies and global surveillance networks, promise to transform our understanding of *M. pneumoniae* as a systemic pathogen and pave the way for evidence-based guidelines, targeted therapies, and preventive strategies.

Future directions for *M. pneumoniae* research span multiple domains: host genetics, immune mechanisms, pathogen virulence, antimicrobial resistance, diagnostics, therapeutics, vaccine development, and collaborative infrastructure. Addressing these knowledge gaps will not only enhance our understanding of extrapulmonary complications but also improve patient outcomes and inform public health strategies globally.

In summary, while many extrapulmonary manifestations of *M. pneumoniae* are reversible, a significant subset can result in long-term morbidity or mortality. The complexity of host–pathogen interactions, variability in clinical presentation, and limitations of current diagnostic and therapeutic approaches underscore the need for ongoing research. Clinicians should maintain a high index of suspicion, particularly in younger patients or those with recent respiratory symptoms and adopt a multidisciplinary approach to management to optimize outcomes. Due to its distinctive clinical profile and the substantial prevalence of extrapulmonary involvement, *M. pneumoniae* infection should be regarded as a systemic disease rather than solely a respiratory illness. Recognition of this broader disease spectrum is essential for optimizing diagnostic strategies and guiding more comprehensive management.

## Figures and Tables

**Table 1:** Extrapulmonary manifestations of *Mycoplasma pneumoniae* infection.

Organ System	Main Manifestations	Proposed Mechanisms	Key Diagnostic Clues	Relative Frequency	Strength of Evidence	Key References
Central nervous system	Encephalitis/meningoencephalitis; ADEM; transverse myelitis; brainstem encephalitis; cerebellitis/ataxia; cranial neuropathies; movement disorders; peripheral neuropathies including GBS variants	Predominantly Indirect (immune-mediated); rarely Direct	MRI: demyelinating lesions; reversible splenial lesion (MERS); CSF lymphocytic pleocytosis ± elevated protein; CSF PCR often negative; temporal association with respiratory illness	Uncommon	Moderate–High for encephalitis/ADEM; Low–Moderate for GBS	[20,21,22,23,24,25,26,27,28,29,30,31]
Cardiovascular	Myocarditis; pericarditis; myopericarditis; arrhythmias; rare intracardiac thrombi	Indirect (immune/cytokine-driven); possible Direct; Vascular occlusion for thrombi	Chest pain/palpitations; troponin/BNP elevation; ECG abnormalities; echo/MRI inflammation	Uncommon	Moderate	[32,33,34,35,36,37,38]
Hematologic	Cold agglutinin AIHA; thrombocytopenia; DIC; transient aPL antibodies	Indirect (autoantibodies/complement activation); Vascular occlusion for thrombosis	Anaemia/jaundice; LDH ↑; indirect bilirubin ↑; DAT positive; cold agglutinin titres; thrombocytopenia/coagulopathy labs	Common (AIHA), Rare (DIC/thrombocytopenia)	High (AIHA); Moderate (other)	[39,40,41,42,43,44,45,46]
Dermatologic/mucocutaneous	Maculopapular rash; urticaria; erythema multiforme; MIRM; SJS; TEN	Indirect (immune-mediated epithelial injury)	Prominent mucositis; biopsy if needed; ophthalmology assessment	Common (rash/MIRM), Rare (SJS/TEN)	High (MIRM/EM); Moderate (SJS/TEN)	[47,48,49,50,51,52]
Musculoskeletal/rheumatologic	Myalgias; arthralgias; reactive arthritis; rare septic arthritis; rhabdomyolysis	Mainly Indirect; rare Direct	Self-limited arthralgia/arthritis; CK elevation in rhabdomyolysis	Common (myalgia/arthralgia), Rare (arthritis/rhabdomyolysis)	Moderate (arthralgia/myalgia); Low (arthritis/rhabdomyolysis)	[53,54,55,56,57,58,59,60]
Renal	Acute glomerulonephritis; interstitial nephritis; nephritic/nephrotic syndromes; AKI	Mainly Indirect (immune complexes/cross-reactive antibodies)	Haematuria/proteinuria; creatinine ↑; complement abnormalities; biopsy in severe cases	Rare	Low–Moderate	[61,62,63,64]
Gastrointestinal/hepatic/pancreatic	Nausea/vomiting/diarrhea; hepatitis; cholestasis; pancreatitis	Mostly Indirect; occasionally unclear	Transaminases ↑; bilirubin ↑; pancreatic enzymes ↑ in pancreatitis	Rare	Low	[65,66,67,68]
Vascular/thrombotic	Venous thrombosis; arterial thrombosis; stroke; limb ischemia; organ infarction; intracardiac thrombosis	Vascular occlusion; may overlap with Indirect immune mechanisms	Imaging-confirmed thrombosis/infarction; D-dimer ↑; aPL positivity (often transient)	Rare	Low–Moderate	[44,69,70,71]
Ocular (selected)	Conjunctivitis; uveitis; optic neuritis	Indirect	Often accompanies mucositis; ophthalmologic evaluation; exclusion of other infectious causes	Rare	Low–Moderate	[20,21,22,23,24,25,26,27,28,47,48,49,50,51,52]
Other/systemic	Fever, fatigue; systemic inflammatory response; rare multi-organ involvement	Mixed (Indirect predominant)	Temporal association with respiratory illness; inflammatory markers; exclusion of other aetiologies	Rare	Low	[3,4,12,13,14,15,16,17,18,19]

Abbreviations: AIHA, autoimmune hemolytic anemia; aPL, antiphospholipid antibodies; ADEM, acute disseminated encephalomyelitis; AKI, acute kidney injury; BNP, B-type natriuretic peptide; CK, creatine kinase; CSF, cerebrospinal fluid; DAT, direct antiglobulin test; DIC, disseminated intravascular coagulation; GBS, Guillain–Barré syndrome; MERS, mild encephalitis/encephalopathy with reversible splenial lesion; MIRM, Mycoplasma-induced rash and mucositis; PE, pulmonary embolism; SJS, Stevens–Johnson syndrome; TEN, toxic epidermal necrolysis.

**Table 2:** Antibiotic treatment recommendations for *Mycoplasma pneumoniae* infections.

Antibiotic	Indication	Main Concerns
Macrolides	Children and adults	Emergence of resistance Drug interactions
Tetracyclines	Older children and adults	Gastrointestinal Tolerability Contraindicated in young children and pregnancy
Fluoroquinolones	Adults	Age-related side effects Contraindicated in young children and pregnancy

## Data Availability

No new data were created or analyzed in this study. Data sharing is not applicable to this article.

## References

[1] Reinmann H. (1938). An acute infection of the respiratory tract with atypical pneumonia: A disease entity probably by a filtrable virus. JAMA.

[2] Waites K.B., Talkington D.F. (2004). *Mycoplasma pneumoniae* and its role as a human pathogen. Clin. Microbiol. Rev..

[3] Narita M. (2010). Pathogenesis of extrapulmonary manifestations of *Mycoplasma pneumoniae* infection with special reference to pneumonia. J. Infect. Chemother..

[4] Bajantri B., Venkatrama S., Diaz-Fuentes G. (2018). *Mycoplasma pneumoniae*: A Potentially Severe Infection. J. Clin. Med. Res..

[5] Gavaud A., Holub M., Asquier-Khati A., Faure K., Leautez-Nainville S., Le Moal G., Goehringer F., Paz D.L., Arnould B., Gerber V. (2025). *Mycoplasma pneumoniae* infection in adult inpatients during the 2023-24 outbreak in France (MYCADO): A national, retrospective, observational study. Lancet Infect. Dis..

[6] Gong C., Huang F., Suo L., Guan X., Kang L., Xie H., Hu G., Yang P., Wang Q. (2024). Increase of respiratory illnesses among children in Beijing, China, during the autumn and winter of 2023. Eurosurveillance.

[7] World Health Organization (WHO) (2023). WHO Statement on Reported Clusters of Respiratory Illness in Children in Northern China.

[8] Meyer Sauteur P.M., Beeton M.L., European Society of Clinical Microbiology, Infectious Diseases (ESCMID) Study Group for Mycoplasma and Chlamydia Infections (ESGMAC), ESGMAC *Mycoplasma pneumoniae* Surveillance (MAPS) Study Group (2024). *Mycoplasma pneumoniae*: Delayed re-emergence after COVID-19 pandemic restrictions. Lancet Microbe.

[9] Uldum S.A., Bangsborg J.M., Gahrn-Hansen B., Ljung R., Molvadgaard M., Fons Petersen R., Wild Svarrer C. (2012). Epidemic of *Mycoplasma pneumoniae* infection in Denmark, 2010 and 2011. Eurosurveillance.

[10] Beeton M.L., Zhang X.S., Uldum S.A., Bébéar C., Dumke R., Gullsby K., Ieven M., Loens K., Nir-Paz R., Pereyre S. (2020). *Mycoplasma pneumoniae* infections, 11 countries in Europe and Israel, 2011 to 2016. Eurosurveillance.

[11] Garzoni C., Bernasconi E., Zehnder C., Frigerio Malossa S., Merlani G., Bongiovanni M. (2024). Unexpected increase of severe *Mycoplasma pneumoniae* pneumonia in adults in Southern Switzerland. Clin. Microbiol. Infect..

[12] Wang Y., Wu L., Chen Z. (2019). The clinical and molecular epidemiology of *Mycoplasma pneumoniae* infections. Int. J. Clin. Exp. Med..

[13] Waites K.B., Balish M.F., Prescott Atkinson T. (2008). New insights into the pathogenesis and detection of *Mycoplasma pneumoniae* infections. Future Microbiol..

[14] Su X., You X., Luo H., Liang K., Chen L., Tian W., Ye Z., He J. (2021). Community-Acquired Respiratory Distress Syndrome Toxin: Unique Exotoxin for *M. pneumonia*. Front. Microbiol..

[15] Xu N., Fan L., Li L., Guo Y. (2024). Exploring the pathogenicity of *Mycoplasma pneumoniae*: Focus on community-acquired respiratory distress syndrome toxins. Microb. Pathog..

[16] Xue G., Zhao H., Yan C., Li S., Cui J., Feng Y., Xie X., Yuan J. (2021). Evaluation of the CARDS toxin and its fragment for the serodiagnosis of *Mycoplasma pneumoniae* infections. Eur. J. Clin. Microbiol. Infect. Dis..

[17] Fernald G.W. (1983). Immunologic mechanisms suggested in the association of *Mycoplasma pneumoniae* infection and extrapulmonary disease: A review. Yale J. Biol. Med..

[18] Narita M. (2016). Classification of Extrapulmonary Manifestations Due to *Mycoplasma pneumoniae* Infection on the Basis of Possible Pathogenesis. Front. Microbiol..

[19] Zuo Y., Zhang R., Li S. (2024). Reviewing advancement in *Mycoplasma pneumoniae* P30 adhesin protein provides insights for future diagnosis and treatment. Front. Microbiol..

[20] Kuwahara M., Samukawa M., Ikeda T., Morikawa M., Ueno R., Hamada Y., Kusunoki S. (2017). Characterization of the neurological diseases associated with *Mycoplasma pneumoniae* infection and anti-glycolipid antibodies. J. Neurol..

[21] Hely M.A., Williamso P.M., Terenty T.R. (1984). Neurological complications of *Mycoplasma pneumoniae* infection. Clin. Exp. Neurol..

[22] Yis U., Kurul S.H., Cakmakci H., Dirik E. (2008). *Mycoplasma pneumoniae*: Nervous system complications in childhood and review of the literature. Eur. J. Pediatr..

[23] Sotgiu S., Pugliatti M., Rosati G., Deiana G.A., Sechi G.P. (2003). Neurological disorders associated with *Mycoplasma pneumoniae* infection. Eur. J. Neurol..

[24] Madzar D., Nickel F.T., Rothhammer V., Goelitz P., Geibdorfer W., Dumke R., Lang R. (2024). Meningitis and intracranial abscess due to *Mycoplasma pneumoniae* in a B cell-depleted patient with multiple sclerosis. Eur. J. Clin. Microbiol. Infect. Dis..

[25] Koskiniemi M. (1993). CNS manifestations associated with *Mycoplasma pneumoniae* infections: Summary of cases at the University of Helsinki and review. Clin. Infect. Dis..

[26] Bonagiri P., Park D., Ingebritsen J., Christie L.J. (2020). Seropositive anti-MOG antibody-associated acute disseminated encephalomyelitis (ADEM): A sequelae of *Mycoplasma pneumoniae* infection. BMJ Case Rep..

[27] Schmuker R.D., Ehret A., Marshall G.S. (2014). Cerebellitis and acute obstructive hydrocephalus associated with *Mycoplasma pneumoniae* infection. Pediatr. Infect. Dis. J..

[28] Hanchosky A.M., Hellmann E., Chong C., Sarvepalli S. (2025). Guillain-Barré Syndrome Induced by *Mycoplasma pneumoniae*: A Non-classical Presentation. Cureus.

[29] Akbar A., Ahmad S. (2021). Atypical case of mild encephalopathy/encephalitis with reversible splenial lesion of the corpus callosum (MERS) associated with *Mycoplasma pneumoniae* infection in a paediatric patient. BMJ Case Rep..

[30] Yuan Z.F., Shen J., Mao S.S., Yu Y.L., Xu L., Jiang P.F., Gao F., Xia Z.Z. (2016). Clinically mild encephalitis/encephalopathy with a reversible splenial lesion associated with *Mycoplasma pneumoniae* infection. BMC Infect. Dis..

[31] Maida E., Kristoferitsch W. (1982). Cerebrospinal fluid findings in *Mycoplasma pneumoniae* infections with neurological complications. Acta Neurol. Scand..

[32] Shefi T.H., Oster Y., Gordon O., Durst R., Michael-Gayego A., Hoss S., Paz R.N., Grupel D. (2025). Cardiac Manifestations of *Mycoplasma pneumoniae* Infections: A Cohort Study 2007–2024. Clin. Infect. Dis..

[33] Paz A., Potasman I. (2002). Mycoplasma-associated carditis. Case reports and review. Cardiology.

[34] Vijay A., Stendhal J.C., Rosenfeld L.E. (2019). *Mycoplasma pneumoniae* Pericarditis. Am. J. Cardiol..

[35] Kenney R.T., Li J.S., Clyde W.A., Wall T.C., O’Connor C.M., Campbell P.T., Van Trigt P., Corey G.R. (1993). Mycoplasmal pericarditis: Evidence of invasive disease. Clin. Infect. Dis..

[36] Balac N., Nelson K.F., Naib T., El-Eshmawi A., Goldman M.E. (2024). The chicken or the egg? *Mycoplasma pneumoniae* complicated by left ventricle thrombus and anterior myocardial infarction: A case report. Eur. Heart J. Case Rep..

[37] Oberoi M., Kulkarni R., Oliver T. (2021). An Unusual Case of Myocarditis, Left Ventricular Thrombus, and Embolic Stroke Caused by *Mycoplasma pneumonia*. Cureus.

[38] Liu J., He R., Wu R., Wang B., Xu H., Zhang Y., Li H., Zhao S. (2020). *Mycoplasma pneumoniae* pneumonia associated thrombosis at Beijing Children’s hospital. BMC Infect. Dis..

[39] Birrow D., Cable R.G., Klein H.G. (1977). Hemolytic anemia with *Mycoplasma pneumoniae* pneumonia. JAMA.

[40] Riad El Khatib M., Lerner A.M. (1975). Myocarditis in *Mycoplasma pneumoniae* Pneumonia: Occurrence with Hemolytic Anemia and Extraordinary Titers of Cold Isohemagglutinins. JAMA.

[41] Tsuruta R., Kawamura Y., Inoue T., Kasaoka S., Sadamitsu D., Maekawa T. (2002). Corticosteroid therapy for hemolytic anemia and respiratory failure due to Mycoplasma pneumoniae pneumonia. Intern. Med..

[42] Thomas M.T., Dennis R.L., Trimble J., Bondranko Z. (2024). A Case of Secondary Autoimmune Hemolytic Anemia Successfully Treated with Rituximab. Cureus.

[43] Bar Meir E., Amital H., Levy Y., Kneller A., Bar-Dayan Y., Shoenfeld Y. (2000). *Mycoplasma-pneumoniae*-induced thrombotic thrombocytopenic purpura. Acta Haematol..

[44] Ascer E., Marques M., Gidlund M. (2011). *M. pneumoniae* infection, pulmonary thromboembolism and antiphospholipid antibodies. BMJ Case Rep..

[45] Miragliotta G., Fumarola D. (1988). Intravascular coagulation and *Mycoplasma pneumoniae* infection. Lancet.

[46] Chryssanthopoulos C., Eboriadou M., Monti K., Soubassi V., Sava K. (2001). Fatal disseminated intravascular coagulation caused by *Mycoplasma pneumonia*. Pediatr. Infect. Dis. J..

[47] Amode R., Ingen-Housz-Oro S., Ortonne N., Bounfour T., Pereyre S., Schlemmer F., Bequignon E., Royer G., Wolkenstein P., Chosidow O. (2018). Clinical and histologic features of *Mycoplasma pneumoniae*-related erythema multiforme: A single-center series of 33 cases compared with 100 cases induced by other causes. J. Am. Acad. Dermatol..

[48] Valle J., Nasrollahi F., Eilbert W. (2022). *Mycoplasma pneumoniae*-induced rash and mucositis. Am. J. Emerg. Med..

[49] McCormack J.G. (1981). *Mycoplasma pneumoniae* and the erythema multiforme—Stevens Johnson syndrome. J. Infect..

[50] Yoosuf F.T., Al Hariri B., Illahi M.N., Sharif M., Yousaf M., Mohamedali M.G., Khalid M.K. (2025). *Mycoplasma pneumoniae*–induced Stevens-Johnson syndrome in an adult: A case report. J. Med. Case Rep..

[51] Terraneo L., Lava S.A.G., Camozzi P., Zgraggen L., Simonetti G.D., Bianchetti M.G., Milani G.P. (2015). Unusual Eruptions Associated with *Mycoplasma pneumoniae* Respiratory Infections: Review of the Literature. Dermatology.

[52] Heymann W.R. (2022). More than Mycoplasma-induced rash and mucositis: The potential role of *Mycoplasma pneumoniae* in Stevens-Johnson syndrome/toxic epidermal necrolysis. J. Am. Acad. Dermatol..

[53] Chu K.A., Chen W., Hsu C.Y., Hung Y.M., Wei J.C.C. (2019). Increased risk of rheumatoid arthritis among patients with *Mycoplasma pneumonia*: A nationwide population-based cohort study in Taiwan. PLoS ONE.

[54] Chu K.A., Chen W., Hung Y.M., Wei J.C.C. (2019). Increased risk of ankylosing spondylitis after *Mycoplasma pneumonia*: A Nationwide population-based study. Medicine.

[55] Marginean C.O., Georgescu A.M., Melit L.E. (2021). Arthritis associated with *Mycoplasma pneumoniae* in a pediatric patient: A case report. Medicine.

[56] Azumagawa K., Kambara Y., Murata T., Tamai H. (2008). Four cases of arthritis associated with *Mycoplasma pneumoniae* infection. Pediatr. Int..

[57] Ponka A. (1979). Arthritis associated with *Mycoplasma pneumoniae* infection. Scand. J. Rheumatol..

[58] Davis C.P., Cochran S., Lisse J., Buck G., Di Nuzzo A.R., Weber T., Reinarz J.A. (1988). Isolation of *Mycoplasma pneumoniae* from synovial fluid samples in a patient with pneumonia and polyarthritis. Arch. Intern. Med..

[59] Gosselt A., Olijhoek J., Wierema T. (2017). Severe asymptomatic rhabdomyolysis complicating a *Mycoplasma pneumonia*. BMJ Case Rep..

[60] Oishi T., Narita M., Ohya H., Yamanaka T., Aizawa Y., Matsuo M., Matsunaga M., Tsukano S., Taguchi T. (2012). Rhabdomyolysis associated with antimicrobial drug-resistant *Mycoplasma pneumonia*. Emerg. Infect. Dis..

[61] Vitullo B.B., O’Regan S., de Chadaverian J.P., Kaplan B.S. (1978). *Mycoplasma pneumonia* associated with acute glomerulonephritis. Nephron.

[62] Carrara C., Abbate M., Sabadini E., Remuzzi G. (2017). Acute Kidney Injury and Hemolytic Anemia Secondary to *Mycoplasma pneumoniae* Infection. Nephron.

[63] Campbell J.H., Warwick G., Boulton-Jones M., McLay A., Jackson B., Stevenson R.D. (1991). Rapidly progressive glomerulonephritis and nephrotic syndrome associated with *Mycoplasma pneumoniae* pneumonia. Nephrol. Dial. Transplant..

[64] Aizawa T., Watanabe S., Tsugawa K., Joh K., Tanaka H. (2021). Membranous nephropathy associated with *Mycoplasma pneumoniae* infection. Pediatr. Int..

[65] Khan H.R.K., Singh A., Usman O., Rafiq S., Amin A. (2022). Acute Pancreatitis: An Unusual Extrapulmonary Manifestation of *Mycoplasma pneumonia*. Cureus.

[66] Sun H., Wang W.Q., Lin L., Shao Z.Y., Zhan L., Tang L.F. (2024). Case Report: *Mycoplasma pneumoniae*-associated acute pancreatitis. Front. Pediatr..

[67] Bi Y., Ma Y., Zhuo L., Yin L., Sheng H., Luan J., Li T. (2021). Risk of *Mycoplasma pneumoniae*-related hepatitis in MP pneumonia pediatric patients: A predictive model construction and assessment. BMC Pediatr..

[68] Romero-Gomez M., Angeles Otero M.A., Sanchez-Munoz D., Ramirez-Arcos M., Larraona J.L., Suarez Garcia E., Vargas-Romero J. (2006). Acute hepatitis due to *Mycoplasma pneumoniae* infection without lung involvement in adult patients. J. Hepatol..

[69] Chiang C.H., Huang C.C., Chan W.L., Chen Y.C., Chen T.J., Lin S.J., Chen J.W., Leu H.B. (2011). Association between *Mycoplasma pneumonia* and increased risk of ischemic stroke: A nationwide study. Stroke.

[70] Sarathchandran P., Al Madani A., Alboudi A.M., Inshasi J. (2018). Mycoplasma pneumoniae infection presenting as stroke and meningoencephalitis with aortic and subclavian aneurysms without pulmonary involvement. BMJ Case Rep..

[71] Yu Z., Meng L., Jia J., Zhang W., Xiong H., Fang F., Li J., Zhuo X. (2025). *Mycoplasma pneumoniae*-Associated Arterial Ischemic Stroke in Pediatric Patients: Clinical Manifestations and Neuroimaging Findings. Pediatr. Neurol..

[72] https://www.cdc.gov/mycoplasma/hcp/clinical-care/index.html.

[73] Kim K., Jung S., Kim M., Park S., Yang H.J., Lee E. (2022). Global Trends in the Proportion of Macrolide-Resistant *Mycoplasma pneumoniae* Infections: A Systematic Review and Meta-analysis. JAMA Netw. Open.

[74] Lai C.C., Hsueh C.C., Hsu C.K., Tsai Y.W., Hsueh P.R. (2024). Disease burden and macrolide resistance of *Mycoplasma pneumoniae* infection in adults in the Asia-Pacific region. Int. J. Antimicrob. Agents.

[75] Li H., Li S., Yang H., Chen Z., Zhou Z. (2024). Resurgence of Mycoplasma pneumonia by macrolide-resistant epidemic clones in China. Lancet Microbe.

[76] Buendia J.A., Patino D.G. (2023). Corticosteroids for the treatment of respiratory infection by *Mycoplasma pneumoniae* in children: A cost-utility analysis. Pediatr. Pulmonol..

[77] Hagman K., Nilsson A.C., Hedenstierna M., Ursing J. (2025). Outcomes of Adjunctive Corticosteroid Treatment in Hypoxemic Adults Hospitalized for *Mycoplasma pneumoniae* Pneumonia: A Retrospective Cohort Study. Clin. Infect. Dis..

[78] Daba M., Kang P.B., Sladky J., Bidari S.S., Lawrence R.M., Ghosh S. (2019). Intravenous Immunoglobulin as a Therapeutic Option for *Mycoplasma pneumoniae* Encephalitis. J. Child Neurol..

[79] Chambert-Loir C., Ouachee M., Collins K., Evrard P., Servais L. (2009). Immediate relief of *Mycoplasma pneumoniae* encephalitis symptoms after intravenous immunoglobulin. Pediatr. Neurol..

[80] Cotter F.E., Bainbridge D., Newland A.C. (1983). Neurological deficit associated with *Mycoplasma pneumoniae* reversed by plasma exchange. Br. Med. J..

[81] Biagi C., Cavallo A., Rocca A., Pierantoni L., Antonazzo D., Dondi A., Gabrielli L., Lazzarotto T., Lanari M. (2021). Pulmonary and Extrapulmonary Manifestations in Hospitalized Children with *Mycoplasma pneumoniae* Infection. Microorganisms.

[82] Poddighe D. (2018). Extra-pulmonary diseases related to *Mycoplasma pneumoniae* in children: Recent insights into the pathogenesis. Curr. Opin. Rheumatol..

[83] Sanchez-Vargas F.M., Gomez-Duarte O.G. (2008). *Mycoplasma pneumoniae*—An emerging extra-pulmonary pathogen. Clin. Microbiol. Infect..

[84] Sondergaard M.J., Friis M.B., Hansen D.S., Jorgensen I.M. (2018). Clinical manifestations in infants and children with *Mycoplasma pneumoniae* infection. PLoS ONE.

[85] Bongiovanni M. (2025). *Mycoplasma pneumoniae* infections: Changing the clinical approach to an evolving disease. Lancet Infect. Dis..

[86] Jiang Z., Li S., Zhu C., Zhou R., Leung P.H.M. (2021). *Mycoplasma pneumoniae* Infections: Pathogenesis and Vaccine Development. Pathogens.

[87] Linchevski I., Klement E., Nir-Paz R. (2009). *Mycoplasma pneumoniae* vaccine protective efficacy and adverse reactions—Systematic review and meta-analysis. Vaccine.

[88] Pan X., Guo X., Shi J. (2024). Design of a novel multiepitope vaccine with CTLA-4 extracellular domain against *Mycoplasma pneumoniae*: A vaccine-immunoinformatics approach. Vaccine.

